# Tooth adornment among siblings living in an urban slum in Nigeria: Health implications for a vulnerable population

**DOI:** 10.1002/ccr3.6563

**Published:** 2023-01-03

**Authors:** Mary E. Osuh, Olaide H. Oyaniran, Tobi S. Tunde‐Alao, Folake B. Lawal, Gbemisola A. Oke, Jackson I. Osuh, Bronwyn Harris, Yen‐Fu Chen, Richard J. Lilford

**Affiliations:** ^1^ Division of Health Sciences Warwick Medical School University of Warwick Coventry UK; ^2^ Department of Periodontology and Community Dentistry Faculty of Dentistry College of Medicine University of Ibadan Oyo Nigeria; ^3^ Department of Periodontology and Community Dentistry Faculty of Dental Surgery University College Hospital Ibadan Oyo Nigeria; ^4^ Department of Psychology Faculty of Social Sciences Federal University Oye‐Ekiti Nigeria; ^5^ Institute of Applied Health Research College of Medical and Dental Sciences The University of Birmingham Birmingham UK

**Keywords:** dental jewelry, fabric stone, rhinestone, super‐glue

## Abstract

Tooth jewelry boosts the appearance and confidence of wearers. However, its use may carry adverse health consequences. This paper creates awareness about a practice not previously reported in Nigeria, to prevent negative health consequences while appropriate measures are taken to quantify and describe the determinants and plan appropriate interventions.

## Graphical abstract

Self‐adornment with dental jewels among youth living in low socioeconomic environments has multiple health implications. Professional dentists are essential to advise, treat, and maintain good oral health in areas where the practice is prevalent.

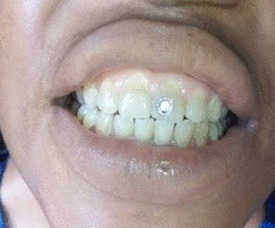

## 1 INTRODUCTION

The use of dental jewelry is an emerging trend in dental cosmetics as it continues to appeal to the fashion sense of today's youth and young adults. Modifications around the mouth and the general body have escalated in proportions in the last 30 years and the use of dental jewelry is relatively new among youths between 18 and 35 years in high‐income countries.^1^, ^2^, ^3^, ^4^ Dental jewelry is considered a status symbol among celebrities, in fashion and in hip‐hop circles in these regions.^1^, ^2^, ^3^, ^4^ There are many forms of dental jewelry: grill jewelry, dazzlers, and twinkles, veneer jewelry, tooth rings, tongue studs, lip studs, lip rings, cheek studs, etc., but the widespread use of jewels fixed to the surface of teeth is a more recent phenomenon. Common as this may seem, however, it has not been reported in the literature nor has it been encountered at the tertiary health facility or the communities where the Principal Investigator works. It was therefore a surprise to see two cases in one setting, raising the consciousness that this may be an emerging trend in Low and Middle‐Income Countries (LMICs).

The stones are commonly used by tailors to embellish fabrics; thus they are readily available in local shops and are quite cheap. Adolescents and youths who follow global fashion trends, emulating celebrities are likely to wear tooth adornment.^5^ From the dental public health perspective, a key issue with dental jewels (rhinestones/twinkles) is that the jewel has to be bonded to the tooth.

This article reports a case of two sisters encountered during a survey, who adorned their teeth with self‐bonded rhinestones. The elder of the sisters was a respondent in a household oral health survey on “Prevalence and Determinants of Oral Diseases and Oral Health Care Needs in a Slum in Nigeria.” The case was an incidental finding in a single site oral health survey,^6^ an extension of the multi‐country survey involving Kenya, Pakistan, Bangladesh, and Nigeria. Improving the health of people living in slums was the main goal of the project and it was sponsored by the National Institute for Health Research Global Health Research Unit, United Kingdom.^7^ Slums are crowded, unhealthy places with high risk of infection and injury. People who live in the slums are often marginalized and have limited access to basic services.^8^, ^9^, ^10^, ^11^

## 2 CASE HISTORY/EXAMINATION

**FIGURE 1:**
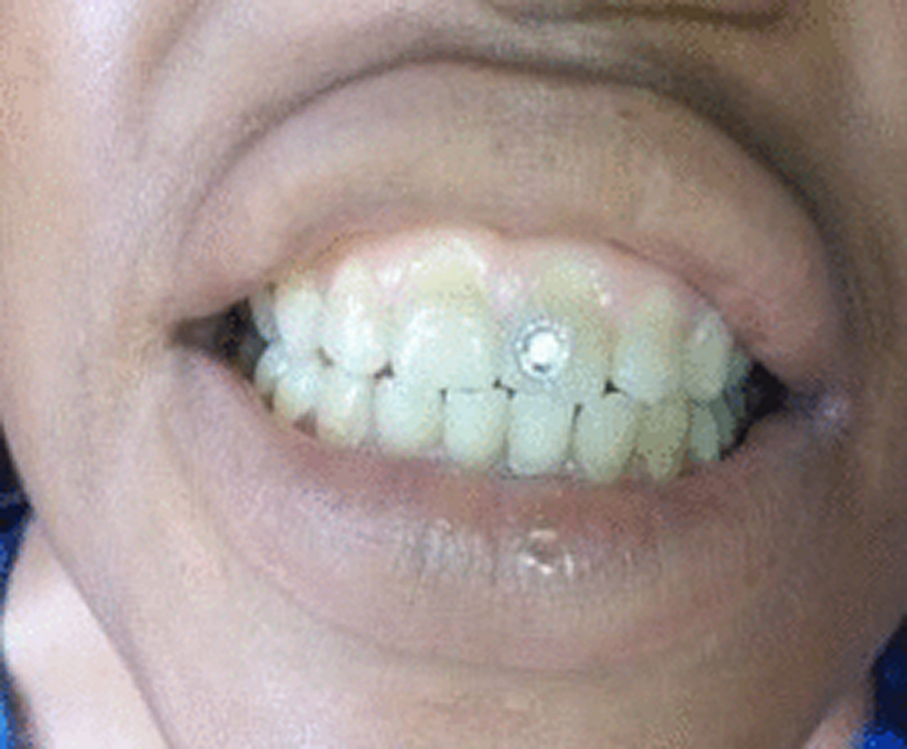
The 21‐year‐old fashion designer wearing a self‐affixed dental stone

The case of a 21‐year‐old female fashion designer who was one of the participants of an oral health survey is presented. Participants for the survey were randomly selected through a multistage sampling technique involving the use of Geographic Information System (GIS), Global Positioning System (GPS), and the random selection algorithm in Open Data Kit (ODK) client application. She had a one‐year history of decorating her upper tooth with self‐bonded stone as was the accepted practice among her peers. She owned a small fashion design business outfit within the community, and lived with her parents, siblings, and extended family in their home. According to her statements, she adorned her tooth for aesthetic reasons as well as to advertise her gemstone trade, which was fast becoming fashion statement in the community. She added that a few of her friends, especially in the fashion design industry also adorned their teeth with gemstones for the same reasons. Gemstones were glued to the upper central incisor teeth using cyanoacrylate adhesive (Superglue, Wenling Aibeisai Adhesive Company). She prematurely de‐bonded her first rhinestone fix, after a week by applying force. Two months later, she re‐fixed another which fell‐off after 2 weeks. Her third attempt was 3 days before our visit. (Figure 1). Asked if she was aware of any health risk involved in the act, she proudly shared her success stories at selling the stones to others.

Intraoral examination revealed a piece of rhinestone bonded to a discolored and rough labial surface of tooth 21. There was increased accumulation of plaque on tooth 21 compared with the adjacent teeth and its plaque index (PI)^12^ score was 1.25. There was mild inflammation of the surrounding gingiva on tooth 21, gingival bleeding score was 0 and periodontal pocket depth was 3.8 mm. Using the simplified Oral Hygiene Index (OHI‐S),^13^ Debris Index (DI‐S) score was 1.17, Calculus index (CI‐S) score was 0.67 and oral hygiene measurement of 1.84 was recorded. This corresponds to a fair oral hygiene level.^13^

**FIGURE 2:**
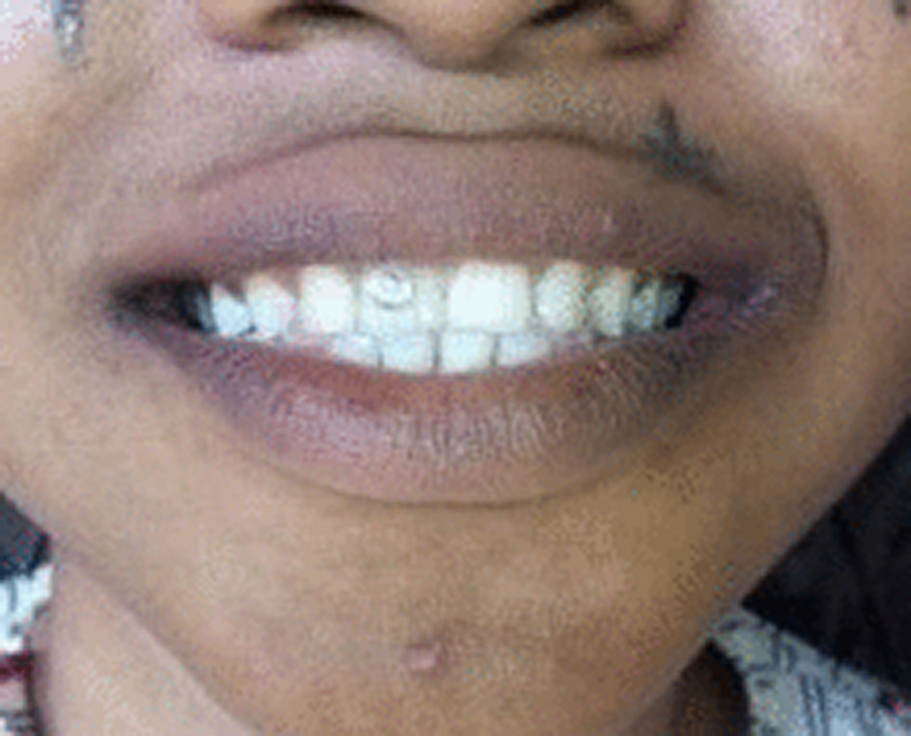
The 18‐year‐old sister to the fashion designer with a similar dental jewelry

The younger sister was simply observing the survey process involving her older sister when a twinkle was observed on her upper incisor tooth (Figure 2). She was 18 years of age and a Senior Secondary School 2 (Grade 2) student in a government secondary school. That was her first experience at adorning the teeth and an attempt to follow the fashion trend like her peers during the summer holidays. Indeed, it was her older sister that applied the first stone for her 3 days before we met her during the household survey. She obviously was not aware of any health risks associated with the practice.

Intraoral examination revealed a rhinestone affixed to a discolored and rough labial surface of tooth 11, increased accumulation of plaque on tooth 11 relative to adjacent teeth, with a plaque index (PI) score^12^ of 1.25. The simplified Oral Hygiene Index (OHI‐S)^13^ value was 1.67 corresponding to a fair oral hygiene level (DI‐S score = 1.17; CI‐S score = 0.50). There was mild inflammation of the surrounding gum (gingivitis), gingival bleeding score was 0 and periodontal pocket depth was 3.5 mm whereas the contralateral tooth had no gingival inflammation, gingival bleeding nor pathologic periodontal pocket.

## 3 OUTCOME AND FOLLOW UP

Follow‐up visits were organized for the two sisters to educate them on the health risks associated with self‐bonding of dental jewels and the importance of consulting a trained dentist for future practices. They were both alarmed when told the possible complications and immediately decided to make a turnaround.

## 4 DISCUSSION

Our case highlights a wave of tooth mutilation which is infiltrating our communities. Clearly, residents have little or no knowledge about the attendant health risks and lack access to professional dental care as they reside in a low resource location.^14^, ^15^, ^16^, ^17^ As it is common practice in such places to self‐medicate,^18^ in this case, they also self‐fix the ornaments. In contrast, the celebrities they emulate abroad often have their stones fixed by the dental professionals.^1^ When professionally applied, rhinestone adornment is non‐invasive, painless and with minimal side effects. It is generally inexpensive but varies according to the jewel applied.^19^, ^20^ Achieving field moisture control, use of the right bonding agent, and the tactile competencies of the dentist determine the success of the application.^1^

In contrast, self‐bonding of rhinestone on the teeth is associated with many health risks which may range from immediate to delayed manifestations.^1^, ^4^, ^21^, ^22^, ^23^

Immediate complications include: accidental dropping and possible aspiration of the stone which can occur during handling and debonding of the stone to the tooth leading to airway obstruction, bleeding, erythema, and endocarditis; others include failure to bond, secondary to poor moisture control; allergic reaction due to the bonding agent or to the presence of foreign material on the tooth, leading to respiratory disturbances; irritation of the soft tissue due to contact of the superglue with the intraoral soft tissue; dermatitis due to contact of the superglue with the skin; and adhesion of the lips and other soft tissues.^1^, ^4^, ^21^, ^22^, ^23^

Delayed complications may include: inability to brush/clean properly; extrinsic discoloration of the tooth; accumulation of plaque and calculus on the tooth in question, promoting poor oral hygiene which may compromise the integrity of the tooth; occurrence of gingivitis from continuous irritation by accumulated plaque and calculus; tooth brushing or biting of sticky foods may de‐bond the attached dental jewelry, the dislodged piece may be aspirated, leading to a medical emergency; direct tissue toxicity and potential for tissue necrosis; decalcification of the affected tooth and subsequent caries formation; and increased susceptibility to periodontal disease.^1^, ^4^, ^21^, ^22^, ^23^

The agent used in the bonding of the rhinestone reported in this article—cyanoacrylate adhesive (superglue, Wenling Aibeisai Adhesive Company), is an all‐purpose essential household adhesive readily available and useful for daily arts and crafts work as it adheres to most surfaces including wood, cardboard, paper, fabric, textiles, glass, cork, straw, leather, China, and pottery.^24^ The cyanoacrylate adhesive group of chemicals have varied toxicity; those used by medical/dental professionals for tissue bonding have lower toxicity but the superglue brand is much more toxic and harmful^25^, ^26^ and may predispose to infection.^27^ Therefore, self‐bonding rhinestone on the teeth using superglue is unsafe. Furthermore, the use of the “do it yourself” factory kits for bonding jewels by laypersons raises questions of moisture control and the quality of the bonding agents^4^ such that the intended result is not achieved along with the attendant risks. Unfortunately, these self‐adhesive kits are inexpensive and readily available in local urban markets in Nigeria, and young persons who purchase them to self‐adorn their teeth may not be aware of the immediate and long‐term adverse effects.

The American Academy of Pediatric Dentistry (AAPD) and the American Dental Association (ADA) both strongly oppose the use of tooth jewelry, due to the complications that can arise from the practice.^28^, ^29^ Since the influence of popular artists on the young, and vulnerable populations may not be so easy to ignore,^30^ safer ways of tooth adornment may be advocated while providing education to enable young people make informed decisions. The AAPD and ADA further recognize the need to educate the public as well as the health professionals on the health implications of tooth jewelry. Raising awareness among public health dentists alongside the general public is of similar importance for planning appropriate intervention programs for young people residing in slums. While the dentists have the responsibility to ensure that the people maintain healthy teeth, they should educate the general public on the dangers of tooth jewelry.^21^ In the event that oral ornaments are to be used, the public should be advised to see their dentist. Regular visits to the dentist, while wearing the dental jewelry should form part of the health education intervention.^1^

### 4.1 Limitation

The authors regret the suboptimal quality of the photographs taken, such that some details could not be clearly seen. The cameras used were limited in quality.

### 4.2 Conclusion

Tooth jewelry is believed to beautify and boost the confidence of the wearer. However, its use is associated with potential complications and therefore should be discouraged. Existing users should be made aware of this and how to prevent them. This case report calls attention to this global trend that is invading our urban communities and the need for prompt intervention to forestall the attendant risks.

## AUTHOR CONTRIBUTIONS

MEO, OHA, and TST wrote the first draft of the manuscript. FBL, GAO, JIO, BH, YFC, and RJL were involved in its critical revision for important intellectual content. All authors discussed and revised the manuscript and gave final approval of the version to be published.

## CONFLICT OF INTEREST

The authors declare that no conflict of interest exists.

## FUNDING STATEMENT

This research was funded by the National Institute for Health Research (NIHR) (NIHR 16/136/87) using UK aid from the UK Government to support global health research. RJL is also funded by NIHR ARC West Midlands. The views expressed in this publication are those of the authors and not necessarily those of the NIHR or the UK Department of Health and Social Care.

## ETHICAL APPROVAL

Ethical approval for the oral health survey from which the cases were observed was obtained from both the Biomedical and Scientific Research Ethics Committee (BSREC) at the University of Warwick, UK (ref. number BSREC37/18–19) and the Oyo State, Nigeria, Research Ethics Review Committee (ref. number‐ AD13/479/1247). A copy each of the ethics approval letters are available for review by the Editor‐in‐Chief of this journal.

## CONSENT

Signed informed consents to use the photos were obtained from the persons involved. A copy each of the signed informed consent forms are available for review by the Editor‐in‐Chief of this journal.

## Data Availability

Data sharing not applicable to this article as no datasets were generated or analysed during the current study.
